# A Randomized, Single-Blind, Placebo-Controlled Study on the Efficacy of the Arthrokinematic Approach-Hakata Method in Patients with Chronic Nonspecific Low Back Pain

**DOI:** 10.1371/journal.pone.0144325

**Published:** 2015-12-08

**Authors:** Akira Kogure, Kazuhiko Kotani, Shigehiko Katada, Hiroshi Takagi, Masahiro Kamikozuru, Takashi Isaji, Setsuo Hakata

**Affiliations:** 1 Physical Medicine and Rehabilitation, Sakura Orthopedic Hospital, Chiba, Japan; 2 Division of Community and Family Medicine, Jichi Medical University, Tochigi, Japan; 3 Department of Public Health, Department of Clinical Laboratory Medicine, Jichi Medical University, Tochigi, Japan; 4 Orthopedics Division, Katada Orthopedic Clinic, Kanagawa, Japan; 5 Department of Orthopedics, Saitama Prefectural Rehabilitation Center, Saitama, Japan; 6 Department of Orthopedics, Oka Orthopedic Hospital, Kanagawa, Japan; 7 Department of Rehabilitation Medicine, Teikyo University School of Medicine, Tokyo, Japan; 8 Physical Medicine and Rehabilitation, Japanese Medical Society of Arthrokinematic Approach, Yamanashi, Japan; Johns Hopkins Bloomberg School of Public Health, UNITED STATES

## Abstract

**Study design:**

cized, single-blind, controlled trial.

**Objective:**

To investigate the efficacy of the Arthrokinematic approach (AKA)-Hakata (H) method for chronic low back pain.

**Summary of Background Data:**

The AKA-H method is used to manually treat abnormalities of intra-articular movement.

**Methods:**

One hundred eighty-six patients with chronic nonspecific low back pain randomly received either the AKA-H method (AKA-H group) or the sham technique (S group) monthly for 6 months. Data were collected at baseline and once a month. Outcome measures were pain intensity (visual analogue scale [VAS]) and quality of life (the Roland-Morris Disability Questionnaire [RDQ] and Short Form SF-36 questionnaire [SF-36]).

**Results:**

At baseline, the VAS, RDQ, and SF-36 scores showed similar levels between the groups. After 6 months, the AKA-H group had more improvement in the VAS (42.8% improvement) and RDQ score (31.1% improvement) than the sham group (VAS: 10.4% improvement; RDQ: 9.8% improvement; both, P < 0.001). The respective scores for the SF-36 subscales (physical functioning, role physical, bodily pain, social functioning, general health perception, role emotional, and mental health) were also significantly more improved in the AKA-H group than in the sham group (all, P < 0.001). The scores for the physical, psychological, and social aspects of the SF-36 subscales showed similar improvement in the AKA-H group.

**Conclusion:**

The AKA-H method can be effective in managing chronic low back pain.

**Trial Registration:**

UMIN Clinical Trials Registry (UMIN-CTR) UMIN000006250.

## Introduction

Chronic low back pain is one of the major causes of health problems. Of all people, 60–85% have low back pain at some time in their lives [1–4]. One study showed that 12–33% of participants reported some back symptoms on the day of the survey, whereas 19–43% reported back pain in the last month, 27–65% in the last year, and 59–84% at some time in their lives [3]. Low back pain can be worse in the long-term, or there may be repeated recurrences [5]. Some reports showed that 75% of patients with low back pain who were seen in general practice still experienced pain 1 year later [6,7]. In Japan, according to the National Livelihood Survey conducted by the Ministry of Health, Labor and Welfare of Japan in 2010, the most common symptom in men was low back pain, and the prevalence was 89.1 per 1,000 people. In women, low back pain was the second most common symptom after shoulder stiffness, and the prevalence was 117.6 per 1,000 people [8].

Various conservative treatments for low back pain have been developed worldwide, and there are several guidelines for treating low back pain in different countries and regions. The use of diagnostic triage for low back pain has been recommended to assess whether there is a specific spinal pathology or nerve root pain and to assess the prognostic factors in combination with the pathophysiology and psychosocial variables [9, 10]. In patients with low back pain but without any features suggesting serious underlying conditions, lumbar imaging may not predict their clinical outcomes [11]. Psychosocial factors are indeed associated with certain clinical forms of low back pain [12–18]. Therefore, it is difficult to determine the best treatment.

The Arthrokinematic Approach-Hakata (AKA-H) method is a manual procedure for treating abnormalities of intra-articular movement of the synovial joint (e.g., joint play, sliding, rolling, and spinning), and it guides movement on the joint surface [19]. The AKA-H method was developed by Setsuo Hakata in 1979 and is based on arthrokinematics [20], articular neurology [21], and the joint mobilization technique [22]. The AKA-H method is being improved to obtain better clinical outcomes, and there are two aspects to this method (i.e., manual medicine and physical therapy) [23,24].

The present study was performed to verify the effects of manual medicine for low back pain. The AKA-H method is not indicated for a specific disease but is indicated for joint dysfunction, arthrogenic pain, joint contractures, and neuromuscular retraining. Hakata et al. demonstrated the efficacy of the AKA-H method in the treatment of acute low back pain [23]. However, the effects of the AKA-H method on low back pain have not yet been studied. Therefore, we conducted a trial to determine the effectiveness of the AKA-H method in adults with chronic low back pain.

## Materials and Methods

### Ethics statement

This clinical trial is registered in UMIN-CTR as R000007397, ID: UMIN000006250, and written informed consent (S1 and S2 Consent Forms) was obtained from the patients. This study was conducted in accordance with the Helsinki Declaration [25] and was approved by Saitama Prefectural Rehabilitation Center’s (SPRC) ethics committee.

### Study patients

Patients with low back pain willing to receive treatment by the AKA-H method called the SPRC first and provided his/her name, address, phone number, and age to the center’s staff. The first author contacted patients by phone within 3 days to provide a full explanation of the randomized controlled trial (RCT). Patients were informed that they may receive the sham technique. Thus, it was promised that those who were randomized to the sham technique would subsequently receive the AKA-H method after completing the 6-month study. Patients who understood the aforementioned explanation, met the inclusion criteria, and agreed to participate in this randomized, controlled trial were consecutively enrolled. We recruited participants from August 30, 2011 and followed them until July 31, 2012.

Low back pain was defined as pain localized from the twelfth rib to the inferior gluteal fold [26]. Nonspecific meant that no specific cause was detectable, such as infection, neoplasm, metastasis, osteoporosis, rheumatoid arthritis, fracture, or radicular syndrome. All patients had a letter of referral and received a definitive diagnosis of chronic nonspecific low back pain from a medical institution (94.2% of the referring physicians were orthopedic specialists who were certified by the Japanese Orthopaedic Association).

The inclusion criteria were as follows: 1) nonspecific low back pain lasting for at least the previous 6 months; 2) the patient received conservative orthopedic treatment from an orthopedic physician or another physician but had no improvement; and 3) the patient’s age was between 18 and 79 years. Patients who underwent surgery of the lumbar spine within the previous 6 months were excluded. In addition, it was assumed that oral medications were continued during the study.

### Study design

The protocol and CONSORT checklist for this trial are available as supporting information (S1 CONSORT Checklist and S1 Protocol). Using a random number table created by free-software R (http://www.r-project.org), patients were randomly assigned to the AKA-H method (AKA-H group) or the sham technique (S group). The eligible patients were relatively uniform, and special consideration was not made for age, sex, or severity. Tasks were performed and confirmed by outpatient nurses who were independent of those who performed the treatment. Authors were not involved in the patient randomization. In addition, treatments were scheduled by appointment only to prevent information from being exchanged among patients. Also, all treatment was performed at the Saitama Prefectural Rehabilitation Center’s outpatient office.

The sham technique was chosen as the control to prove that the effectiveness of the AKA-H method was not just a placebo effect. Similarly, a comparison between acupuncture and the sham technique was conducted to prove its efficacy by Vickers et al. [27].

**Fig 1:**
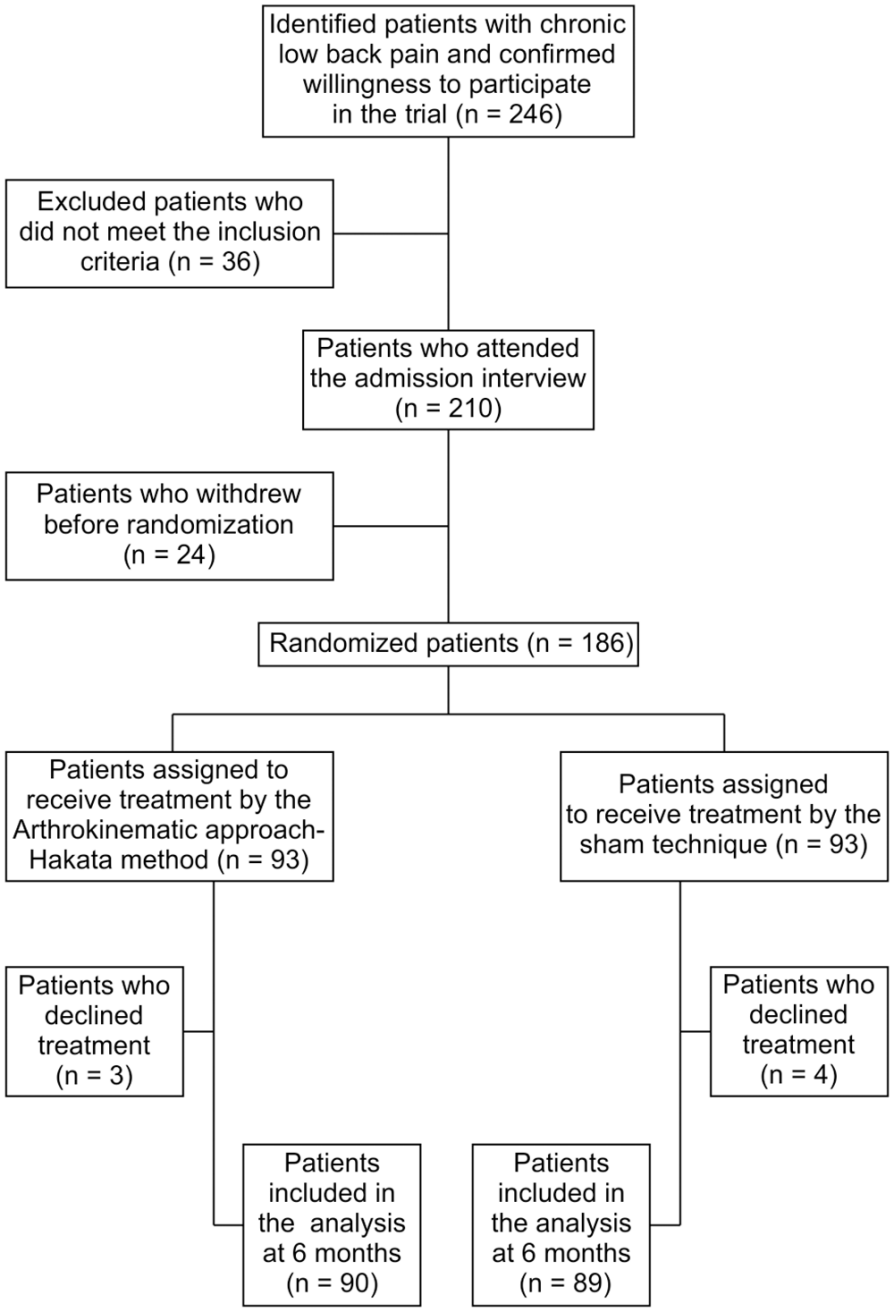
Study flow diagram.

This study was a parallel group comparison in which the AKA-H group and S group were compared for 6 months. The study was randomized at the level of the individual patient, and it was a single-blind test in which only patients were blinded to the details. The advantage of this single-blind study is that by blinding participants, researchers are more likely to avoid potentially biased responses. The disadvantage of this single-blind study is that an experimenter risks consciously or subconsciously affecting subjects’ responses. The study flow chart is depicted in Fig 1.

### Interventions

**Fig 2:**
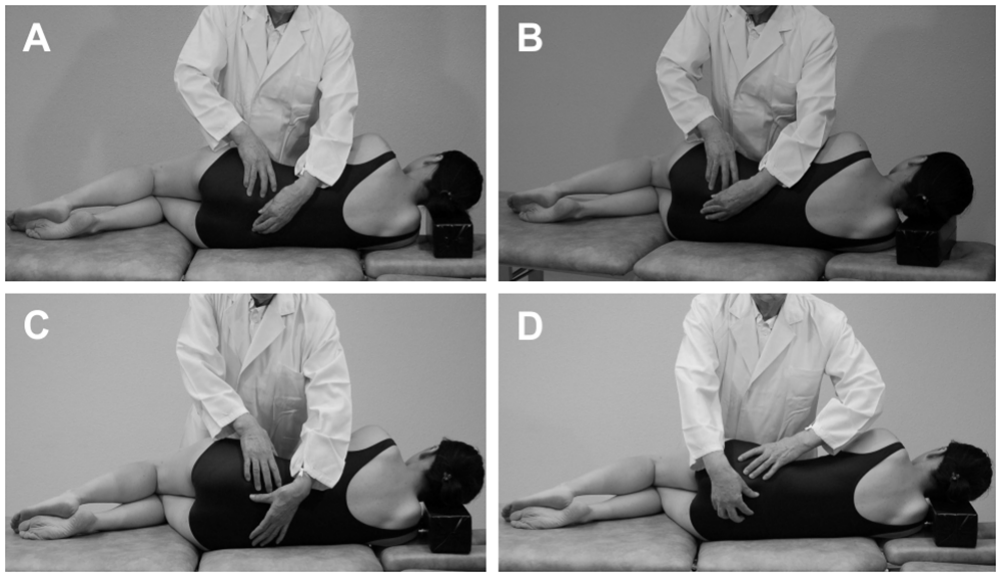
The four techniques of the Arthrokinematic approach-Hakata method. **(A)** Upward gliding (**B)** downward gliding **(C)** superior distraction **(D)** inferior distraction.

Interventions were only conducted by the first author, a physician certified as a qualified preceptor by the Japan Medical Society of the Arthrokinematic Approach. Patients were randomized to receive the AKA-H method or sham technique. The AKA-H method was applied once a month for 6 months to the sacroiliac joint for treatment of low back pain using the following techniques: 1) upward gliding, 2) downward gliding, 3) superior distraction, and 4) inferior distraction (Fig 2).

Patients were asked to lie on his/her side with the upside being treated, and the therapist stood in front of the patient. In upward gliding, the left index finger was placed on the S1 spinous tubercle, and the proximal phalanx of the right thumb was placed on the highest position of the iliac crest with palmar abduction of the right thumb. The right ring finger glided from the caudal side with the radial side of the ring finger pressed hard on the posterior superior iliac spine, pushing into the cranial direction, and when movement of the sacroiliac joint was felt, the S1 spinous tubercle was pushed in the cranial ventral direction using the left index finger. In downward gliding, the same procedure for upward gliding was performed until movement of the sacroiliac joint was felt. Then the S3 spinous tubercle was pushed in the caudal direction using the left index finger. In superior distraction, the left thumb was lightly placed on the S1 spinous tubercle, and the proximal phalanx of the right thumb was placed on the highest position of the iliac crest with palmar abduction of the right thumb. Next, the distal phalanx of the right ring finger was placed on the posterior superior iliac spine, the position was shifted about 1 cm to the cranial side, and from there, the hand slid in the caudal direction. When the proximal phalanx of the thumb hit the iliac crest and the ulnar side of the ring finger hit the posterior superior iliac spine, the fingertips were pressed down hard to stop the sliding. Then the force was applied to either the right ring finger or the thumb to pull the ilium in the longitudinal direction of the therapist’s right forearm. In inferior distraction, the right thumb was lightly placed on the S3 spinous tubercle, the left thumb was placed on the anterior superior iliac spine with palmar abduction, and the left ring finger was placed on the posterior superior iliac spine. The position of the left hand was shifted about 1 cm to the caudal side, and from there, the left hand slid in the cranial direction. When the radial side of the distal phalanx of the left thumb hit the anterior superior iliac spine and the ulnar side of the distal phalanx of the left ring finger hit the posterior superior iliac spine, the fingertips were pressed down hard to stop the sliding. Then the force was applied to either the left thumb or the ring finger to pull the ilium in the longitudinal direction of the therapist’s left forearm.

During the sham technique, the patient was in the side-lying position with both hips and knees extended (each joint was close-packed), and the therapist stood on the ventral side of the patient. The therapist’s thumb, on the cephalic side of the patient, was placed on the S1 spinous tubercle, giving a light downward force while the index finger of the therapist’s other hand was placed underneath the S1 spinous tubercle to counteract the downward force; thus, the sacroiliac joint did not actually move. The same procedure was performed in which the therapist’s thumb, on the caudal side of the patient, was placed on the S3 spinous tubercle with the index finger of the therapist’s other hand underneath it. This procedure was slowly repeated twice with the procedure on S1 first, followed by S3, alternately. Because AKA-H is manual therapy for gently and slightly moving the sacroiliac joint, a warm-up is not necessary. Both techniques took about 15–20 minutes to complete.

### Outcomes

Outcomes were assessed using the visual analogue scale (VAS) [28], Japanese version of the Roland-Morris Disability Questionnaire (RDQ) [29–31], and Japanese version of the 36-Item Short-Form Health Survey (SF-36) [32–36]. The main outcome measure was the average level of low back pain on the VAS during the last 30 days of the 6-month treatment (0 represents no pain and 100 represents the maximum pain imaginable). Although there are studies that doubt its validity [37,38], the VAS is the most frequently used indicator for evaluating pain, and it was adopted as a main outcome measure because its validation has been evaluated [39,40]. Patients were asked to record the VAS scores using a specialized scale (S1 and S2 Figs) three times a day at approximately the same time each day: in the morning, at noon, and in the evening. A dedicated calendar, designed for recording the VAS scores three times per day (S3 Fig), was mailed to patients at least 1 month prior to the date of the first visit. The average value of the VAS scores for 30 days before the start of treatment was considered the pre-treatment VAS score. The VAS score at each month was calculated as the average value of the VAS scores on the previous 30 days. The average value during 1 month was used, because the study subjects were patients who had chronic low back pain over a long period, and changes in the VAS score were not very large. Therefore, the average value during 1 month was evaluated as a representative value.

Patients were also asked to complete the RDQ and SF-36 in the waiting room prior to each treatment. The nurse checked for any omissions, and if descriptions were missing, patients were required to complete their answers.

The RDQ is a scale that allows patients to assess their degree of disability experienced during daily activities due to low back pain. Since it is generally difficult to compare the RDQ by using raw scores, a comparison was made using the deviation score method with reference to the mean values by age and sex in Japanese individuals [41–44]. The deviation score was determined from the following formula: Deviation score = Mean score of the RDQ in patients with low back pain for that month—The obtained RDQ score in patients for that month ÷ Standard deviation of the RDQ’s normative value × 10 + 50.

In the deviation score method, the mean score was set at 50 points, and the standard deviation was set at 10. Therefore, it was easy to determine where the RDQ score of a patient was in the distribution of the RDQ scores for the entire patient population. This was the advantage of this method [29–31].

The SF-36 questionnaire is frequently used to evaluate one’s general quality of life (QOL) [32–36]. This questionnaire measures health-related functioning in eight subscales: physical functioning (PF), role limitations due to physical problems (RP), bodily pain (BP), vitality (VT), general health perceptions (GH), social functioning (SF), role limitations due to emotional problems (RE), and mental health (MH).

### Sample size determination

Considering changes in the VAS score (as a main outcome) in the AKA-H (the experimental arm) and S groups (the independent control arm), differences in the VAS score between groups was 10 (mean level) with a standard deviation of 23 (based on our preliminary experience). Therefore, we needed to recruit 84 participants in each group with an **α** of 5% and power of 80%. Assuming a dropout of <10%, we set the total sample size to 186 participants.

### Statistical analysis

Data are presented as mean ± standard deviation. Differences between data at baseline between groups were analyzed using the unpaired t and chi-square tests. Since rare dropout cases (3 in AKA-H group and 4 in Sham group) existed and most cases did not dropout easily, missing data were analyzed by applying the list-wise case deletion method. Additionally, last observation carried forward was performed (S1 Table). A two-way (time period and treatment group) repeated measures analysis of variance was used to observe differences in data between groups during the intervention period. Regarding multicomparison between groups at each time point, we used the Bonferroni method. SPSS, version 20.0 (SPSS, Inc., Chicago, IL, USA) was used for all the statistical analyses. A P-value <0.05 was considered statistically significant.

## Results

Among 246 eligible patients with low back pain, 210 attended the admission interview between August 30, 2011 and July 31, 2012. Of 210 patients, 24 did not consent to randomization and declined to participate in the study after the first visit to our outpatient office. Therefore, 186 patients agreed to participate in this study.

There were 3 dropouts in the AKA-H group (disappearance of symptoms: n = 1, dissatisfaction with treatment: n = 1, and transfer to another hospital: n = 1). In the sham group, there were 4 dropouts (lost contact: n = 2, transferred to another hospital: n = 1, and dissatisfaction with treatment: n = 1). Therefore, the analysis was done with 90 research participants from the AKA-H group and 89 from the sham group, totaling 179 patients.

Mean age of 179 subjects was 59.8 ± 13.1 years. The majority were women (62.0%), and we used Asian BMI classification to represent patients’ degree of obesity [45]. Mean duration of chronic low back pain before visiting our center was 59.9 ± 63.8 months, and mean level of low back pain at baseline was 52.9 ± 18.8 on the VAS. All patients had already consulted a physician, mostly an orthopedic surgeon (93.2%), for their low back pain. This study did not have any criteria for VAS scores at baseline for study entry. There was no statistical impact regarding the baseline characteristics and outcomes (i.e., the VAS, RDQ, and SF-36) (Table 1).

**Table 1:** Baseline demographics and clinical characteristics of the study participants.

	**Total**	**AKA-H group**	**Sham group**	**P-value**
	(n = 179)	(n = 90)	(n = 89)	
**Demographics**				
Age (years)	59.8 ± 13.1	60.0 ± 12.7	59.6 ± 13.3	0.857*
Sex (female/male, %)	62.0/38.0	60.0/40.0	64.0/36.0	0.527^+^
Body mass index (kg/m^2^)	23.2 ± 5.1	23.7 ± 5.4	22.6 ± 4.7	0.260*
Education duration (years)	13.4 ± 3.2	13.6 ± 3.4	13.2 ± 2.9	0.414*
Living with someone/alone (%)	86.6/13.4	86.7/13.3	86.5/13.5	0.926^+^
**Physical demands of employment (%)**				
No employment	17.9	16.7	19.1	0.871^+^
Sedentary work	9.5	10.0	9.0	0.806^+^
Non-sedentary	35.2	35.6	34.8	0.949^+^
**Clinical factors**				
Medication use (%)	100	100	100	1.0^+^
Duration of low back pain (months)	36.0 [12.8–87.0]	36.0 [12.0–120.0]	42.0 [15.0–72. 5]	0.681^c^
Surgery recommendation (%)	22.3	22.2	22.4	0.948^+^
Clinic visits for low back pain (times)	5.4 ± 3.2	5.2 ± 3.5 (1–14)	5.6 ± 2.7 (1–13)	0.376*
Pain intensity (visual analogue scale)	52.7 ± 18.8	54.5 ± 18.2 (11.9–95.3)	50.8 ± 19.3 (8.8–97.8)	0.169*
Disability (RDQ: deviation score)	38.2 ± 9.5	37.6 ± 10.2 (4.7–56.2)	38.8 ± 8.8 (12.0–59.0)	0.488*
**SF-36 (points)**	**Total**	**AKA-H group**	**Sham group**	**P-value**
Physical functioning	30.1 ± 14.0	30.1 ± 14.9 (-8.2–55.1)	30.0 ± 13.0 (-8.2–55.1)	0.943*
Role physical	28.9 ± 13.3	28.2 ± 13.2 (1.7–56.2)	29.6 ± 13.3 (1.7–56.2)	0.566*
Bodily pain	31.8 ± 6.7	31.5 ± 6.7 (17.2–49.0)	32.0 ± 6.6 (17.2–49.0)	0.569*
Social functioning	33.4 ± 12.8	34.5 ± 13.1 (4.5–57.1)	32.3 ± 12.5 (11.1–57.1)	0.468*
General health perception	38.0 ± 8.1	38.2 ± 9.6 (11.4–64.2)	37.8 ± 6.6 (20.8–57.0)	0.967*
Vitality	39.6 ± 8.7	39.2 ± 8.1 (22.6–56.4)	40.0 ± 9.2 (19.5–59.5)	0.662*
Role emotional	35.0 ± 12.5	35.1 ± 13.6 (5.6–56.6)	34.9 ± 11.4 (5.6–56.6)	0.814*
Mental health	41.2 ± 8.9	41.8 ± 9.0 (19.9–62.4)	40.6 ± 8.8 (19.9–62.4)	0.374*

Data are shown as mean ± standard deviation, median [IQR], or percentages. RDQ, Roland-Morris Disability Questionnaire; SF-36, 36-Item Short-Form Health Survey; AKA-H, Arthrokinematic approach Hakata method. BMI is defined as the weight in kilograms divided by the square of the height in meters (kg/m^2^). The AKA-H group was treated using the AKA-H method, whereas the sham group (control) was treated using the sham method.

* Unpaired t-test;

^+^ chi-square test;

^c^ Mann-Whitney U test.

**Fig 3:**
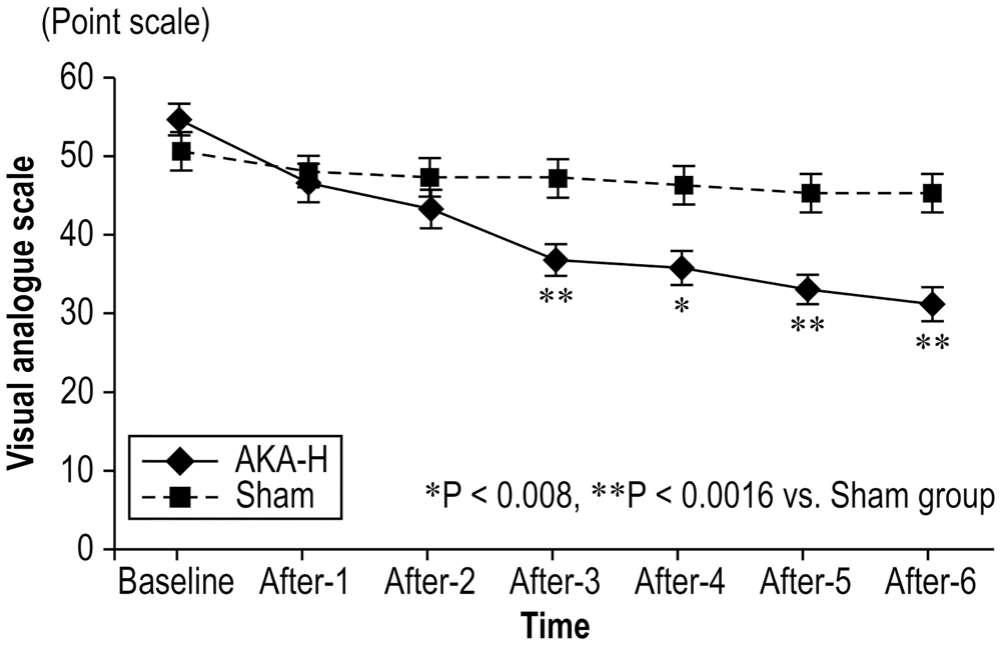
Comparison of the average level of low back pain between the AKA-H and sham groups. * Statistical significance between the AKA-H and sham groups after treatment (P < 0.05: two-way [group and month] analysis of variance). Data are expressed as means and standard error of the mean. AKA-H, Arthrokinematic Approach-Hakata.

Regarding outcomes every month after starting treatment, there were no significant differences in the VAS score between groups for the first 2 months. However, from the third month, the VAS score was significantly reduced in the AKA-H group compared to the sham group (Table 2, Fig 3).

**Table 2:** Levels of pain, disability, and quality of life at 1 month to 6 months after treatment (list-wise case deletion method).

	1 month		2 months		3 months		4 months		5 months		6 months		P-value
													[time and group]
	AKA-H group	Sham group	AKA-H group	Sham group	AKA-H group	Sham group	AKA-H group	Sham group	AKA-H group	Sham group	AKA-H group	Sham group	
	mean ± SD	mean ± SD	mean ± SD	mean ± SD	mean ± SD	mean ± SD	mean ± SD	mean ± SD	mean ± SD	mean ± SD	mean ± SD	mean ± SD	
	(n = 90)	(n = 89)	(n = 90)	(n = 89)	(n = 90)	(n = 89)	(n = 90)	(n = 89)	(n = 90)	(n = 89)	(n = 90)	(n = 89)	
VAS (points)	46.8 ± 20.6	47.5 ± 20.5	43.4 ± 20.0	47.6 ± 21.1	36.8 ± 17.8^††^	47.4 ± 21.2	36.0 ± 17.4^††^	46.3 ± 22.2	33.0 ± 17.6^††^	45.6 ± 21.9	31.2 ± 18.8^††^	45.5 ± 22.0	<0.001
RDQ (deviation score)	41.7 ± 11.0	41.1 ± 9.5	43.3 ± 10.9	41.5 ± 9.8	44.7 ± 10.4	41.8 ± 9.7	47.0 ± 9.2^†^	42.5 ± 10.1	47.8 ± 9.7^††^	42.6 ± 9.8	49.3 ± 9.3^††^	42.6 ± 9.9	<0.001
SF-36 (points)													
Physical functioning	33.2 ± 14.3	31.7 ± 13.5	35.7 ± 13.0	30.8 ± 13.5	37.8 ± 12.8^††^	31.3 ± 13.5	40.0 ± 12.6^††^	32.2 ± 13.7	40.9 ± 13.5^††^	32.3 ± 14.0	41.8 ± 14.0^††^	32.5 ± 14.3	<0.001
Role physical	31.7 ± 12.9	32.3 ± 13.3	34.7 ± 12.4	32.5 ± 13.5	36.1 ± 12.9	31.8 ± 14.6	38.7 ± 12.5^††^	31.9 ± 14.4	40.7 ± 12.7^††^	32.3 ± 13.6	40.6 ± 13.4^††^	32.2 ± 13.5	<0.001
Bodily pain	34.7 ± 7.3	34.0 ± 6.9	37.0 ± 7.2^†^	34.0 ± 7.3	37.8 ± 8.1^†^	34.2 ± 7.1	39.8 ± 8.0^††^	34.5 ± 7.4	41.3 ± 9.0^††^	34.3 ± 7.4	42.2 ± 9.7^††^	33.5 ± 7.7	<0.001
Social functioning	37.9 ± 12.7	35.4 ± 12.2	40.1 ± 12.6	35.7 ± 12.1	42.6 ± 11.7^††^	34.0 ± 12.6	45.5 ± 11.2^††^	34.8 ± 12.6	45.9 ± 11.2^††^	35.9 ± 12.3	48.0 ± 10.7^††^	34.6 ± 12.9	<0.001
General health perception	40.0 ± 9.9	39.5 ± 6.4	40.6 ± 9.9	39.9 ± 6.6	42.7 ± 8.8^†^	39.4 ± 7.3	43.4 ± 9.3^†^	39.7 ± 7.9	43.9 ± 9.0^††^	39.4 ± 7.4	44.7 ± 9.8^††^	39.3 ± 7.8	<0.001
Vitality	42.0 ± 8.8	42.2 ± 9.0	43.9 ± 9.2	42.3 ± 9.3	45.9 ± 9.1^†^	41.5 ± 10.1	46.8 ± 9.2^††^	41.3 ± 10.4	47.6 ± 9.2^††^	41.0 ± 10.0	47.9 ± 9.7^††^	41.5 ± 10.0	<0.001
Role emotional	37.3 ± 13.4	37.6 ± 11.3	40.0 ± 11.9	37.0 ± 11.3	41.6 ± 11.7^†^	36.5 ± 12.7	44.8 ± 11.0^††^	36.5 ± 12.3	45.3 ± 12.0^††^	36.7 ± 11.9	45.8 ± 11.9^††^	35.8 ± 12.0	<0.001
Mental health	43.8 ± 9.2	42.2 ± 8.4	45.6 ± 9.7^†^	41.8 ± 8.2	47.4 ± 9.4^††^	42.2 ± 8.4	48.7 ± 9.0^††^	41.6 ± 8.8	48.6 ± 10.5^††^	41.7 ± 9.6	49.6 ± 9.7^††^	42.0 ± 8.8	<0.001

VAS, visual analogue scale; RDQ, Roland-Morris Disability Questionnaire; SF-36, 36-Item Short-Form Health Survey; AKA-H, arthrokinematic approach Hakata method. Significant difference (two-way [time period and group] repeated measures analysis of variance). AKA-H vs. sham group using Bonferroni multiple comparison tests at each time point:

^†^ < 0.008,

^††^ < 0.0016.

Two questionnaires also provided evidence for the efficiency of the AKA-H method in the treatment of low back pain. The RDQ (deviation score) scores were significantly higher in the AKA-H group than in the sham group from the fourth month (Table 2). The SF-36 revealed significant benefits after 2 months for bodily pain and mental health. After 3 months for physical functioning, social functioning, general health perception, vitality, and role emotional and after 4 months, all scores were significantly higher in the AKA-H group than in the sham group (Table 2). These data show that the AKA-H method successfully addressed all aspects of low back pain.

### Side effects

Side effects such as low back pain and muscle weakness were reported by 8 patients in the AKA-H group and by 10 in the S group. However, symptoms were mild in all patients. These side effects improved within 2–3 days post-treatment, and patients continued participation (Table 3).

**Table 3:** Side effects experienced by study subjects.

Signs/symptoms	AKA-H group (n = 90)	Sham group (n = 89)
Low back pain	4 (2.2 days)	5 (1.5 days)
Muscle weakness	2 (2.5 hours)	3 (2 day)
Lower leg numbness	2 (1.5 day)	2 (2 days)

AKA-H, Arthrokinematic approach Hakata method. Data shown are the number of participants who experienced side effects followed by the average duration of the side effect in parentheses.

## Discussion

The present study found that the AKA-H method improved pain intensity and the QOL in patients with chronic nonspecific low back pain to a greater degree than the sham technique during a 6-month period. Until recently, there has been little evidence on the efficacy of this treatment, although the AKA-H method has been used for treating low back pain in Japan and the Japanese Medical Society of Arthrokinematic Approach is acknowledged as a member of the International Federation for Manual/Musculoskeletal Medicine [46]. Since many suffer from low back pain and the treatments are often difficult to manage [1–8], these findings are relevant to the field of pain control medicine. In addition, although the AKA-H method is still not always a prevalent treatment, the number of physicians who want to learn the AKA-H method has recently increased steadily. Thus, the results may stimulate interest from these physicians and raise awareness about the treatment.

There are many conservative treatments for chronic nonspecific low back pain such as pharmacological treatments [47–49], cognitive behavioral therapy [50,51], attendance at back schools [52,53], exercise therapy [54,55], physical therapy [56], manipulation [57–59], and massage [60–62]. However, conflicting claims exist for nearly every form of conservative therapy used to manage low back pain [63,64]. For instance, a comparative study on the efficacy among physical therapy, chiropractic manipulation, and the provision of educational booklets for treating patients with low back pain concluded that any of these treatments showed only a small effect on low back pain [65]. There is a lack of evidence that manual therapy is superior to other treatments, yet there is evidence on the efficacy of physical therapy for low back pain [66]. van Middelkoop et al. [67] discussed the efficacy of various physical and rehabilitation interventions with respect to low back pain in a systematic review and found a low overall evidence level and low evidence levels in efficacy comparisons between exercise therapy and usual care, and between behavioral therapy and no treatment. Only in the case of multidisciplinary treatment were acts such as no treatment and active treatment shown to exhibit moderate evidence of effective pain reduction, despite being short-term. Additionally, they were unable to gain complete data from treatments such as back schools, low-level laser therapy, patient education, massage, traction, superficial heat/cold, and lumbar supports because of issues such as heterogeneity of the populations.

Alternatively, an additional study reported that a significant improvement was seen in the group of patients who practiced an exercise program such as increasing muscle strength and stretching as instructed by medical staff at an outpatient clinic, compared to the group treated with non-steroidal anti-inflammatory drugs (NSAIDs) [68]. This study also indicated that the reason why therapeutic interventions for low back pain did not show clear outcomes may be the fact that pain expression is different in various countries and that patients’ cultural diversity may also have some influence. For example, the economic situations of patients have a partial influence on whether patients with low back pain seek treatment [69]. In addition, efforts have been made recently to improve patients’ outcomes further by classifying chronic low back pain. However, a method of classification that can be adopted for all purposes does not yet exist [70].

According to the European guidelines for the management of chronic nonspecific low back pain, cognitive-behavioral treatment, multidisciplinary interventions, noradrenergic or noradrenergic-serotonergic antidepressants, muscle relaxants, NSAIDs, and weak opioids are recommended as treatments [71]. Guidelines for the evaluation and management of low back pain have been established by the American Pain Society [72,73].

There are at least 11 international guidelines for low back pain, and most have similar statements regarding the recommended treatments, although there are some differences regarding exercise therapy, spinal manipulation, and the use of muscle relaxants [72,73]. This difference may be due to different interpretations of the evidence and different levels of importance placed on the benefits to patients, side effects, and costs in different countries. There is an opinion that most of these guidelines focus on the treatment of acute low back pain but do not provide specific guidelines for managing chronic low back pain [74]. Accordingly, effective conservative treatments remain largely inconclusive [71–74].

From the results of the present study, the AKA-H method can be a useful tool for treating chronic low back pain. In addition, there were no specific side effects associated with the AKA-H method. The occurrence rates of the side effects were at least similar to that of the sham technique. Our findings further indicate the usefulness of administration of the AKA-H method in the clinical setting.

The underlying mechanism of the efficacy of the AKA-H method for low back pain remains unclear in our study. We know that the possibility of impairment to the intra-articular movements, which is referred to as joint dysfunction [75], has some influence on the occurrence of low back pain; however, the reason why this impairment causes pain has not been clarified. It is thought that joint dysfunction may stimulate type four articular receptors by increasing tension of the articular capsule ligaments [21]. Future studies on the biological mechanisms responsible for the present findings are required.

As for the effectiveness of a labor-intensive (1:1) approach such as the AKA-H method for chronic low back pain, an asymmetrical relationship can occur, in which the doctor is the provider and the patient is the dependent, and this may result in a sick-role dependency. This seems to be similar to the condition of acute low back pain. Even when using the RCT methodology, it is necessary to be aware of drawbacks such as this. However, although there is a lack of detailed description in the methods, the effectiveness of AKA-H for patients with acute low back pain is suggested in this same way as in the present research. The same type of treatment may be effective for patients with acute and chronic pain, and this can be considered an advantage of this approach.

Our study has limitations. First, the doctor was not blinded to the treatment, which was impossible to accomplish in this study. Second, this study was performed at only one hospital in Japan, and we did not invite the public to participate. Generalization of the results is limited; therefore, collaborative research at multiple centers should be conducted in the future. With regard to the statistical analyses, there is a possibility that better results could have been obtained if a third group (i.e., a waiting list only or normal treatment group) was added for comparison between groups. However, participants of this study were patients who had already suffered from chronic low back pain for a long period with no effect from common treatment methods. Additionally, randomizing individuals to a group that would receive no treatment or only the common treatment would discourage participation. Third, most patients (62%) were women, but in this study, the results were not assessed according to sex differences. Women have been reported to note pain more emphatically than men [76], and a previous report indicated that the placebo effect is larger in women than in men in terms of medical treatments [77]. Thus, whether the physician’s sex would have a bearing on the results is a topic worthy of further study. Fourth, there may be a type I error due to the multiple comparisons; however, all measurements showed the same trend between groups after 6 months, so it appeared that there was validity in our findings. Fifth, the sham group had been promised to receive AKA-H 6 months later. No patients knew which treatment they were receiving because they had not experienced the AKA-H method before they were recruited into the study. The Hawthorne effect [78] is the process in which human subjects of an experiment change their behavior and response because of some expectation. We acknowledge that this bias may have influenced the results of this study. Finally, although the AKA-H approach has been shown to significantly improve the functional outcomes of chronic low back pain, further research should consider the incorporation of exercise therapy [67] and behavioral interventions [51] to comprehensively address long-term improvement of this chronic condition.

## Conclusions

Although there are many limitations, the present study found that the AKA-H method significantly improved pain intensity and the QOL in patients with chronic nonspecific low back pain during a 6-month period. This suggests that the AKA-H method can be effective in managing chronic low back pain.

## Supporting Information

S1 CONSORT Checklist CONSORT Checklist for randomized trials. (PDF)

S1 Consent Form Informed consent from patient 1. (PDF)

S2 Consent Form Informed consent from patient 2. (PDF)

S1 Fig Specialized scale for the visual analogue scale scores. (TIF)

S2 Fig Specialized scale for the visual analogue scale scores (Japanese). (TIF)

S3 Fig Dedicated calendar for patients to record visual analogue scale scores three times per day. (TIF)

S1 Protocol Trial Protocol. (DOCX)

S2 Protocol Trial Protocol in Japanese. (DOCX)

S1 Table Levels of pain, disability, and quality of life at 1 month to 6 months after treatment (LOCF). Data are shown as mean ± standard deviation. VAS, visual analogue scale; RDQ, Roland-Morris Disability Questionnaire; SF-36, 36-Item Short-Form Health Survey; AKA-H, Arthrokinematic approach Hakata method. Significant difference (two-way [time period and group] repeated measures analysis of variance): * P < 0.05, ** P < 0.01. The AKA-H vs. sham group using Bonferroni multicomparison tests, † P < 0.008, †† P < 0.0016.
(DOCX)
