# Supplementary material for: A Randomized, Single-Blind, Placebo-Controlled Study on the Efficacy of the Arthrokinematic Approach-Hakata Method in Patients with Chronic Nonspecific Low Back Pain
Source: PLoS One. 2015 Dec 8;10(12):e0144325. doi: 10.1371/journal.pone.0144325 (PMC4672908; doi:10.1371/journal.pone.0144325)
Supplement: S1 Consent Form — (PDF) [file pone.0144325.s002.pdf]

PLoS PUBLIC LIBRARY of SCIENCE

Consent Form for Publication in a PLoS Journal

I, the undersigned, give my consent for my photograph and/or case history to be published in a Public Library of Science (PLoS) Journal. I have seen and read the material to be published. I have discussed this consent form with Akira Kogure who is an author of this paper, and I understand the following:

All PLoS journals are freely available on the web[1]. Hence, anyone anywhere in the world can read material published in them. Readers include not only doctors, but also journalists and other members of the public.

My name will not be published, and as far as possible all identifying features will be removed. However, it is not possible to ensure complete anonymity, and someone may be able to recognize me.

I understand that under the license which the PLoS uses (the Creative Commons Attribution License[2]) material published in PLoS journals can be redistributed freely and used for any legal purpose, including translation into other languages and commercial uses. I also understand that signing this consent form does not remove my rights to privacy.

Name SETSUO HAKATA

Date May 21, 2015

Signed Setso Hakata

Author AKIRA KOGURE

Date May 21, 2015

Signed Akira Kogure

[1]PLoS Journals: http://www.plos.org/journals/

[2]Creative Commons Attribution License: http://creativecommons.org/licenses/by/2.5/

Please complete this form, obtain the patient's signature, and file in case notes.

The manuscript reporting this patient's details should state that consent to publication was obtained from the patient.
