# Supplementary figures and images for: A Randomized, Single-Blind, Placebo-Controlled Study on the Efficacy of the Arthrokinematic Approach-Hakata Method in Patients with Chronic Nonspecific Low Back Pain
Source: PLoS One. 2015 Dec 8;10(12):e0144325. doi: 10.1371/journal.pone.0144325 (PMC4672908; doi:10.1371/journal.pone.0144325)

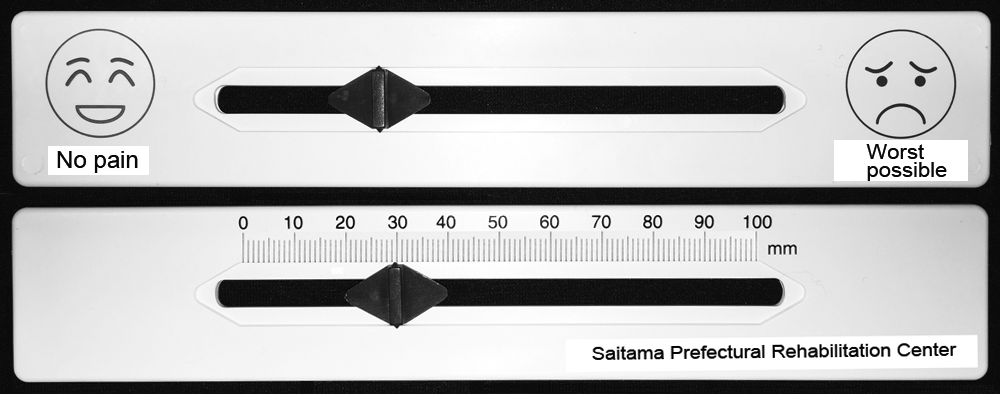

Supplement: S1 Fig — (TIF) [file pone.0144325.s004.tif]

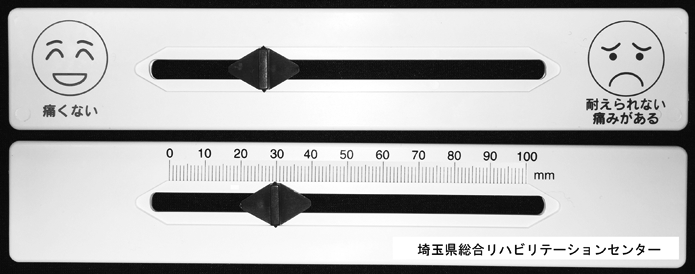

Supplement: S2 Fig — (TIF) [file pone.0144325.s005.tif]

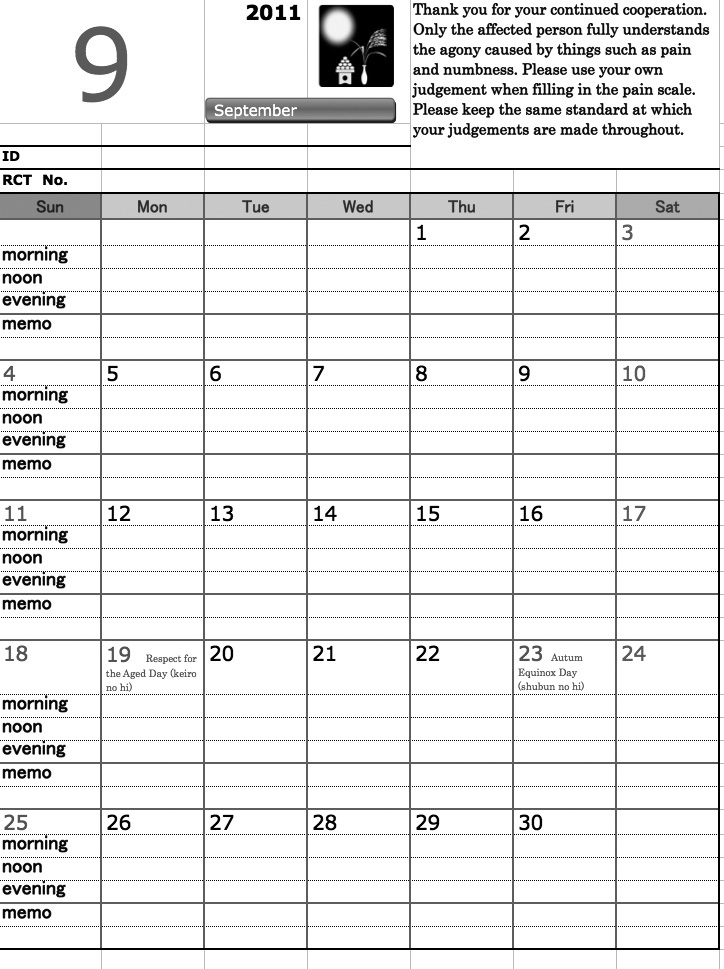

Supplement: S3 Fig — (TIF) [file pone.0144325.s006.tif]
