# Supplementary material for: A Randomized, Single-Blind, Placebo-Controlled Study on the Efficacy of the Arthrokinematic Approach-Hakata Method in Patients with Chronic Nonspecific Low Back Pain
Source: PLoS One. 2015 Dec 8;10(12):e0144325. doi: 10.1371/journal.pone.0144325 (PMC4672908; doi:10.1371/journal.pone.0144325)
Supplement: S1 Protocol — (DOCX) [file pone.0144325.s007.docx]

| **Arthrokinematic approach-Hakata method for Low Back Pain: a Randomized Controlled Trial**  (This paper is registered on this UMIN. The registration number is UMIN000006250 and the registration date is August 30th, 2011)  **Clinical Trial Protocol**  Location of Research: Department of Rehabilitation, Saitama Prefectural Rehabilitation Center, Saitama, Japan  **Chief Investigator** Akira Kogure  E-mail: [anakin_kogure@ybb.ne.jp](mailto:anakin_kogure@ybb.ne.jp); Telephone: +81-048-781-2222  **Trial Design** Shigehiko Katada  Email: [katada@aroma.ocn.ne.jp](mailto:katada@aroma.ocn.ne.jp); Telephone: +81-465-36-7590  **Trial** **Consulting** Setsuo Hakata  Email: [tubaki@sakai.zaq.ne.jp](mailto:tubaki@sakai.zaq.ne.jp); Telephone: +81-722-98-4740  **Co-investigator** Kazumasa Kotani  Email: [kazukotani@jichi.ac.jp](mailto:kazukotani@jichi.ac.jp); Telephone: +81-285-58-7386 |
| --- |

**1. Rationale & background information**

Chronic lower back pain is associated with high morbidity worldwide, and a report suggests that more than half the world population experiences lower back pain at least once in their lifetime. Many patients with non-specific chronic lower back pain are not indicated for surgical procedure and are treated by conservative therapy. To date, sufficiently satisfactory approaches to treat lower back pain have not been determined. The Arthrokinematic Approach-Hakata (AKA-H) method is an approach uniquely developed in Japan to manually treat abnormal intracapsular movement. The aim of this study was to demonstrate that this approach can be an effective therapy for non-specific chronic lower back pain.

**2. Study goal and objective**

**Study goal and objective:** To determine the effectiveness of AKA-H method for chronic lower back pain.

**3. Study Design**

・Using a random number table created by free-software R (http://www.r-project.org), the patients were randomly assigned to the AKA-H (AKA-H) or the sham (S) groups.

・This study was a parallel group comparison in which the AKA-H group and S group were compared for 6 months. The study was randomized at the level of the individual patient, and it was a single-blind RCT in which only the patients were blinded to the details.

**4. Methodology**

**・Patient selection criteria**

The subjects of this study were individuals who met all of the following inclusion criteria and for whom none of the following exclusion criteria apply.

**Inclusion Criteria:**

・Non-specific lower back pain lasting for at least the previous 6 months

・The patient received conservative orthopedic treatment from an orthopedic physician or another physician but had shown no improvement in symptoms

・The patients’ age was between 18–79 years.

**Exclusion Criteria:**

- History of spinal operations within the 6 months before the trial.
- Current history of infection, neoplasm, metastasis, osteoporosis, rheumatoid arthritis, fracture, or radicular syndrome
- Pregnancy

**Sampling and Recruitment**

AKA-H has been widely known among patients in Japan through mass communication or personal communication and is expected to be referred from home doctors. A list of hospitals where AKA-H is available is shown on the website of the Japanese Medical Society of Arthrokinematic Approach(http://www.aka-japan.gr.jp). Doctors listed there are specialists or advising doctors who have passed an exam stipulated by the Society. When patient desires treatment by AKA-H, he/she will be required to call Saitama Prefectural Rehabilitation Center first, and will be asked his/her address, phone number, and age by a Center staff. Then, the first author will contact him/her by phone within 3 days to give a full explanation of the RCT. If the patient shows his/her willingness to participate in the study, he/she will receive an email with instructions on the AKA-H procedure, consent form, treatment flow at the first visit, specially created calendar type form to record VAS scores, and a scale to measure VAS.

**・Randomization**

Output 0 or 1 in order using a random number table created free-software R (for example, 0 for the first person, 1 for the second person, and 1 for the third person). A piece of paper with 0 on it is enclosed in an envelope with 1 on it, and the envelope is sealed. A piece of paper with 1 on it is enclosed in an envelope with 2 on it, and then the envelope is sealed. This procedure will be done by a nurse in the outpatient department. The nurse will keep and store the envelopes without being informed of the purpose of creating those envelopes so that the first author cannot be involved in the randomization. At the patient’s first visit, a nurse will bring an envelope with the number corresponding to the patient number to the first author after the patient walks in the outpatient medical examination room. Then, the first author opens the envelope to provide sham procedure or AKA-H after confirmation of the number without informing the patient. Caution is needed to have the patient not recognize which procedure will be performed.

**Treatment schedules**

**Duration and Frequency**

- Patients will be treated only as outpatients.
- Outpatient treatment shall be once a month for six months, with evaluation in the seventh month.
- In principle, the oral medication will be continued.

**Interventions**

The patients are randomized to receive the AKA-H method or the sham technique. The AKA-H method is applied once a month for 6 months to the sacroiliac joint for treatment of low back pain using the following techniques: 1) upward gliding, 2) downward gliding, 3) superior distraction, and 4) inferior distraction.

- **Sham technique:** During the sham technique, the patient lies sideways with both hips and knees extended (each joint is close-packed), and the therapist stands on the ventral side of the patient. The therapist’s thumb, on the cephalic side of the patient, is placed on the S1 spinous tubercle, giving a light downward force while the index finger of the therapist’s other hand is placed underneath the S1 spinous tubercle to counteract the downward force; thus, the sacroiliac joints do not actually move. The same procedure is performed in which the therapist’s thumb, on the caudal side of the patient, is placed on the S3 spinous tubercle with the index finger of the therapist’s other hand underneath it. This procedure is slowly repeated twice with the procedure on S1 first, followed by S3, alternately.

**Change criteria: Combination therapy**

- If patient so wishes, a disclosure is given immediately, and Sham procedure can be changed to AKA-H method. A combination treatment is possible, with continuous administration of both the oral medicine and the analgesic patch.
- In the event that lower back pain ceases completely, and that patient does not wish to have treatment, this constitutes a complete recovery. In the event that patient wishes to terminate treatment or if deterioration of general condition of patients or the presence of an illness involving the vital prognosis is suspected, therapy is suspended immediately, and a thorough examination and appropriate treatment is performed.

**Post-Treatment Therapy**

Once the test is complete, disclosure is made to the patients as to whether they had AKA-H treatment or Sham treatment, and patients are asked if they wish to continue treatment. If treatment was Sham, patients were allowed to change treatment to AKA, and if treatment was AKA-H, patients could continue with AKA-H.

**Safety Considerations**

Facilities in every area of Japan are using this treatment method, and although these facilities are not great in number, there have been no reports of any serious adverse events to the treatment as of yet.

**Possible Adverse Events**

- Mild low back pain or lower leg numness is reported; patient is to report immediately by phone if this type of symptom occurs, and is recommended to come in to the hospital as soon as possible.
- Patient is examined as needed upon arrival at hospital, continuation of treatment is determined based on consultation with patient.

**Reporting Procedures for Adverse Events**

In the event of any serious adverse events, the person in charge of the research shall report to the head of the hospital and to the AKA Medical Society, and appropriate treatment shall be taken.

**5. Outcomes and Confidentiality**

Primary outcome and secondary outcomes

- Primary outcome is mean VAS score over a month from the previous visit.
- Participants are required to record their VAS score in a form every day at almost the same time for at least 1 month until the first visit and bring the recorded form along with consent form at the first visit.
- Secondary outcomes are Roland-Morris Disability Questionnaire (RDQ) and 36-Item Short-Form Health Survey (SF-36).
- Participants will fill RDQ and SF-36 at every visit including the first visit at the waiting room by advance reservation only before seeing a doctor. Participants are required to visit the outpatient office once a month, in principle, for 6 months after initiation of treatment.
- A VAS record form will be collected at every visit, and a new one will be provided to the participants.
- RDQ and SF−36 will be filled at the waiting room and will be collected at every visit. Those collected record forms will be stored in the locked locker at outpatient department.

**6. Statistical Analysis**

- All statistical analyses were performed using the principle of intention-to-treat analysis.
- For missing data, the data were analyzed by extrapolating the last observation.
- A two-way (time period and treatment group) repeated measures analysis of variance will be used to observe the difference in data between the two groups during the intervention period.

**7. Ethics**

**Protection of the Patients**

The study will be performed in accordance with the ethical principles in the Declaration of Helsinki.

**Informed consent for patients**

Prior to registration for the trial, the attending physician will provide patients with an informed consent form that meets the approval of the ethics committee of the Saitama Prefectural Rehabilitation Center, explain the tests sufficiently as dictated by the needs of the individual patient, and obtain the patients’ agreement in writing that they are participating in the trial of their own free will.

The informed consent form shall be signed or sealed and dated by the responsible research physician who provided the explanation. The physician in charge of the research shall provide patients with a copy of the signed or sealed and dated informed consent form with explanatory materials, and attach a copy of the form to their patient record prior to the patient's participation in the trial. The informed consent form shall be retained for one year.

Consent may be withdrawn at any time and may, as a general rule, be communicated by the patient himself or herself to the responsible physician during examinations or via phone call.

**Privacy Policy**

Identification and referral of registrants were performed through a randomly assigned registration number assigned at the time of registration. Personal data such as names, birth dates, addresses, phone numbers, et cetera are not shared or disclosed by the medical institution.

**Approval by Saitama Prefectural Rehabilitation Center Ethics Committee**

Prior to implementation of these trials and throughout the time scheduled for implementation of the trials, approval shall be obtained from the Saitama Prefectural Rehabilitation Center Ethics Committee for the implementation and continuation of the trials from the perspective of ethical, scientific, and medical propriety and validity. Research representatives shall submit to the Saitama Prefectural Rehabilitation Center Ethics Committee documents for its review, including trial implementation schedules, informed consent forms, etc.

**Changes to Protocol Contents**

In accordance with "9. Changes to Protocol Contents," when making changes to contents of the protocol, it will be necessary to submit an application for revision to the Ethics Review Board and to gain their approval.

**8. Expense Allocation & Compensation**

- Funding and Financial Relationships

There is no funding for the treatment, and there are no conflicts are interest (COIs).

- Expense Allocation for Testing

The clinical testing has no source of funding, so the subjects will be covered under their usual health insurance.

- Compensation for Health Damage and Insurance

Compensation for health damage shall be through the medical malpractice insurance of the physician in charge of research.

- Liability Insurance

The physician in charge of research shall have medical malpractice insurance. Subjects whose health has been damaged by researchers shall be receive in good faith the appropriate treatment as covered by health insurance.

**9. Changes to Protocol Contents**

In the event of changes to contents of the implementation planning document, it is necessary to submit a "written application for changes in protocol contents" to the Ethics Review Board of Saitama Prefectural Rehabilitation Center and to gain its approval beforehand.

In the event that the protocol is altered, physicians responsible for conducting the study will revise the explanatory documents given to subjects based on the changes.

**10. Termination and Early Discontinuance of Testing**

Early discontinuance of testing is considered to be a situation where a portion or the entirety of the testing is discontinued ahead of schedule for any one of the following reasons:

1. The effectiveness or ineffectiveness of the treatment being tested is verified at an early stage.
2. It is determined early on that there is little likelihood of proving the effectiveness of the treatment being tested.
3. It is determined that there is a problem with the safety of the treatment being tested or the control treatment, based on an event report of a seriously harmful illness or on data outside of the aforementioned clinical testing.

**11. Preservation of Records**

The data collected shall be stored until the research representative completes this study or until at least one year to the day from the completion of the study. The medical institution conducting the study shall retain records for the longest period of time authorized (items such as medical examination and treatment records) along with documents stored at the medical institution conducting the study (items such as plans for implementing the study and informed consent documents).

**12. Attribution of Findings and Publication**

- UMIN Clinical Trials Registry, etc. will be conducted based on directives from ICMJE.
- Biometricians and data managers will be included as collaborators during the announcement of results.
- Provisions for the order of authors according to the order of the number of registered medical cases as well as provisions for corresponding authors will be conducted in advance.

**13. Appendix files**

- Pain scale and Recording paper
- Description document and consent form
