# Supplementary material for: A Randomized, Single-Blind, Placebo-Controlled Study on the Efficacy of the Arthrokinematic Approach-Hakata Method in Patients with Chronic Nonspecific Low Back Pain
Source: PLoS One. 2015 Dec 8;10(12):e0144325. doi: 10.1371/journal.pone.0144325 (PMC4672908; doi:10.1371/journal.pone.0144325)
Supplement: S2 Protocol — (DOCX) [file pone.0144325.s008.docx]

| 慢性腰痛に対するAKA-博田法の有効性に関する臨床試験  （臨床試験登録番号：UMIN000006250）  **Clinical Trial Protocol**  研究代表者  埼玉県総合リハビリテーションセンター  リハビリテーション科  木檜晃（こぐれあきら）  E-mail : [anakin_kogure@ybb.ne.jp](mailto:anakin_kogure@ybb.ne.jp); 電話：048-781-2222  臨床試験デザイ  **ン**片田重彦  E-mail : katada@aroma.ocn.ne.jp; 電話：+81-465-36-7590  コンサルティング 博田節夫  E-mail : tubaki@sakai.zaq.ne.jp; 電話：+81-722-98-4740  共同研究者 小谷和彦  E-mail : kazukotani@jichi.ac.jp; 電話：+81-285-58-7386 |
| --- |

1. **論拠と背景**

人生のうちで一度でも腰痛を経験する人は半数以上という報告があり、世界中で慢性腰痛に悩む人は多い。非特異性慢性腰痛に対する治療は手術の適応がないことが多く、保存療法が行われるが、その効果のほどは高くない。そのなかでも、十分な研究はなされていないが一つの有効な治療手段として、AKA-博田法と呼ばれる治療法が存在する。この治療法は関節包内運動を徒手的に改善するものであり、当研究はその有効性を科学的に検証するものである。

**2. 研究のゴールと目的**

- 研究のゴール：当研究にてその有効性が示されれば、AKA-博田法の普及につながり、患者さんのベネッフィトとなりうる。
- 目的：非特異性慢性腰痛に対するAKA-博田法の有効性を示す。

1. **研究デザイン**

- 非特異性慢性腰痛患者(patients with chronic non-specific low back pain)はAKA-Hakata

methodによる治療を受ける群(AKA-H group)とSham procedure を受ける群(S group)かのいずれかに、ソフトのR で作成した乱数表を用いてランダムに割り当てられる。

**・**当研究はAKA-H groupおよび S groupの両群を６ヶ月間平行して比較する平行群間比較であ

り、個人レベルでランダム化され、試験参加者のみがブラインド化される単盲検RCTである。

1. **方法論**

**・　患者選択規準**

　　以下の適格規準を全て満たし、かつ以下の除外規準のいずれにも該当しない患者を、本研究

　　 の対象患者とする。

**適格規準**

　・非特異的慢性腰痛を持ち6ヶ月以上腰痛が持続している患者。

　・整形外科またはかかりつけ医師に保存的治療を受けたが効果がなかった患者。

　・18歳から79歳までの男性患者及び女性患者。

**除外規準**

　　　・過去6カ月以内に脊椎の手術を受けた患者。

　　・感染、悪性腫瘍、悪性腫瘍転移、骨粗鬆症、関節リウマチ、骨折、神経根障害による腰痛

　　のある患者。

　　　　・妊婦

**・　サンプリングと参加者の募集**

　　日本におけるAKA-Hは患者間ではマスコミや口コミを通じてかなり知られるところとなっており、ま

　　た、かかりつけ医からの紹介も見込まれる。AKA医学会のサイト(http://www.aka-japan.gr.jp)には治

　　療が受けられる病院一覧が開催されておりそこに掲載されている医師は、AKA医学会の定める試験

　　に合格した専門医、および指導医である。AKA-Hによる治療を希望する人は、まずSaitama

　　Prefectural Rehabilitation Centerに電話をして、本人の住所、電話番号、年齢を教えてもらうが、こ

　　の対応はセンター職員が行う。続いて3日以内に、first author が本人へ電話で連絡を取り、RCTの

　　内容を十分な時間をかけて行う。そこで研究参加の意志のある人に対して、AKA-H手技の説明及

　　びRCTに関する同意書や初診日の治療に関する流れ、VAS記載のために特別に作られたカレンダ

　　ー式の用紙とVASを計るための定規を郵送する。

・　**割り付け**

free-software Rにて作成した乱数表を用いて0または1の数字を順番に打ち出す（例えば、1番目は0、2番目は1、3番目は1など）。そして1番と書いた封筒の中に0と書いた紙を入れのりで封をする。2番と書いた封筒には1と書いた紙を入れてのりで封をする、というように外来部門の看護師が作成する。その際、この封筒は何に使うのかを看護師に知らせずに封筒を作成し、その後これらの封筒は外来看護部門に管理、保存され、first author が関われないようにする。患者の初診時、外来診察室に患者が入室後、患者番号と同じ番号の着いた封筒を看護師がfirst authorまで運び、first authorが開封、数字を確認し、shamまたはAKA-Hを患者に告げずに行う。その際、どちらの手技を行っているのかを患者がわからないように注意する。

- **治療計画**

**頻度、期間**

　　　　　・治療対象は外来患者のみに限定。

　　　　　・外来での治療は1ヶ月に1回とし計6回治療を行い7ヶ月目に評価する。

　　　　　・原則として内服薬は継続とする。

**治療の実際**

患者はAKA-H法とSham手技のいずれかに無作為に振り分けられる。AKA-H法は月１回づ

　　　　　つ６ヶ月間続けられ仙腸関節にアプローチする。通常4つのテクニックがあり、1)上方滑, 2)下方

　　　　　滑り、3)上部離開、4)下部離開である。

・　Sham手技：両側の股関節、膝関節を伸展位、すなわちCPP(close-packed position)とし、

　　　右側臥位の場合であれば、術者は患者の腹側に立ちS１の棘結節の左側、すなわち天

　　　井方向から左母指でベッド側へ、S１の棘結節の右側すなわちベッド側から右示指を使っ

　　て、そのカウンターの役目を、次にS３棘結節に対しては、左母指の代わりを右母指で、右

　　示指の代わりを左示指で、同様の操作を行う。すなわち、極力仙腸関節を動かさないように

　　これらの手技を施行することが重要で、その回数はS1、S3ともに、天井側から押す指を交

　　互にかえたものをワンセットとして、これを２セット、ゆっくり繰り返す。

変更規準、併用療法

・患者の希望があれば開示を即座に行い、Sham手技であった場合はAKA-博田法の治

　療に変更することができる。

　　　・併用療法として内服薬及び湿布薬は許容される。

　　　　治療中止規準、完了規準

　　　　　　・腰痛が全くなくなった場合で患者が治療を希望しない場合は治療完了とみなす。

・患者が治療中止を希望する場合または患者の全身状態の悪化や生命予後に関わる疾

　患の存在が疑われる場合は治療を直ちに中止し、精査、治療を行うものとする。

**治療終了後の治療**

　試験終了時点でAKA-博田法であったかShamであったかを、患者本人に開示し治療

　の継続希望を聞く。Shamであった場合、希望があればAKA-博田法による治療に変更す

　ることができる、またAKA-博田法であった場合そのまま継続ができる。

**安全注意事項**

　　　　　　 当治療法については、施設数は多くはないが、日本各地で行われており、重篤な有害

　　　　　　　事象は今までに報告はない。

**予期される有害事象**

- ・軽度の腰痛、下肢のしびれなどが報告されており、生じた場合は患者に電話で直ち

　　　　　　に報告してもらい、可能な限り早期の来院を促す。

　　　　　　　・来院時点で必要な診察検査を行い、治療継続の有無を患者とともに決定する。

**有害事象の報告と対応**

　　　　・万が一重篤な有害事象が生じた場合は、研究責任者が、医療機関の長、AKA医学

　　　　　会へ報告し、　適切な対処を行う。

**5. Outcomeと守秘義務**

**主要評価項目及び副次評価項目**

**・primary outcomeは**visual analogue scale (VAS)の1ヶ月間の平均とする。

・短くとも最初の治療日より１ヶ月以上前から毎日、朝、昼、夕、ほぼ同一時刻に専用スケールにて

計測し、当院から郵送された専用カレンダーに本人が記載し、外来治療のたびに持参してもらう。

　　　・Secondary outcomeはRoland-Morris Disability Questionnaire (RDQ) 及び36-Item Short-Form

　Health Survey (SF-36)とする。

- 参加者は初診日を含めて、完全予約制の待合室にて受診前にRoland-Morris Disability

Questionnaire (RDQ)および36-Item Short-Form Health Survey (SF-36)の記載を行う。受診は原則1ヶ月に1回の割合で、外来にて治療開始後6ヶ月間続けられる。

・ ＶＡＳを記載した用紙は外来受診ごとに回収し、新たなものが患者に渡される。RDQ と

SF−36については、患者は受診時に待合室で記入し、その都度回収する。回収された記

録用紙は外来看護部門の鍵のかかったロッカーで管理される。

**6. 統計学的事項**

・統計解析はITT解析を行い、欠損値処理法はLOCF法(last observation carried forward)に従う。

・AKA-博田法を行ったグループとSham手技を行ったグループの有効性比較では２元配置分散

　分析（時間×治療）を用いる。

**7. 倫理的事項**

**患者の保護**

本試験はヘルシンキ宣言に基づく倫理的原則を遵守して実施する。

**患者への説明と同意（インフォームド・コンセント）**

担当医は、患者が本試験に参加する前に、埼玉県総合リハビリテーションセンター倫理委員会で承認の得られた同意説明文書を用いて、患者本人に十分に説明し、本試験への参加について自由意志による同意を文書により得るものとする。

・同意書には説明を行った研究責任医師が記名捺印又は署名し、各自日付を記入する。研究責任医師は、患者が本試験に参加する前に、記名捺印又は署名と日付が記入された同意書の写し及び説明文書を患者に渡し、同意書をカルテに保管するものとする。同意書の保管期間は１年間とする。

・同意撤回はいつでも可能で、その旨電話または受診時に原則本人が担当医師に伝えるものとす

　る。

**プライバシーの保護**

登録者の同定や紹介は、登録時に割り当てられた登録番号（randomization時に割り当てられた番号）を用いて行われる。登録患者の氏名、生年月日、住所、電話番号等の個人データは実施医療機関から他へもれることはない。

**埼玉県総合リハビリテーションセンター倫理委員会による承認**

本試験実施前及び試験実施予定期間中を通じて、埼玉県総合リハビリテーションセンター倫理委員会において、本試験の実施、継続等について倫理的、科学的及び医学的妥当性の観点から承認を得るものとする。研究代表者は、実施計画書、説明同意文書など審査の対象となる文書を埼玉県総合リハビリテーションセンター倫理委員会に提出する。

**プロトコルの内容変更について**

プロトコルの内容を変更する際には、「9. プロトコルの内容変更」に従い、倫理審査委員会に改訂の申請を行い、承認を得る必要がある。

**8. 費用負担と補償**

**資金源及び財政上の関係**

治療の資金源はなく、利害の衝突conflict of interest: COIもない。

**試験にかかる費用負担**

・臨床試験に係わる資金源がないため、通常の保険診療内で被験者が負担する。

**健康被害の補償及び保険への加入**

**健康被害の補償**

補償は研究責任医師の医師賠償保険による。

**補償・賠償保険への加入**

・研究責任医師は医師賠償保険に加入している。

・研究者から健康被害を受けた被験者には健康保険適応治療として、誠実に対応する。

**9. プロトコルの内容変更**

実施計画書の内容を変更する場合には、変更に先立ち、「プロトコルの内容変更申請書」を埼玉県総合リハビリテーションセンター倫理委員会に提出し、承認を得る必要がある。

・プロトコルに改定があった場合には、試験責任医師は、それに応じて被験者への説明文書を改

　定する。

**10. 試験の終了と早期中止**

・試験の早期中止とは、以下のいずれかの理由により予定よりも早く試験全体または一部が中止されることを指す。

　１）試験治療の有効性における優位性または劣性が早期に確認された。

　２）試験治療の優位性を証明できる可能性が小さいことが早期に判明した。

　３）重篤な有害事象報告又は当該臨床試験以外の情報に基づき、試験治療または対照治療の安全性に問題があると判断された。

**11. 記録の保存**

収集したデータについては研究代表者が本試験終了、もしくは中止後最低1年が経過した日まで保管するものとする。原資料（診療記録等）ならびに試験実施医療機関で保管される書類（実施計画書、同意説明文書等）については、実施医療機関が許可する最長期間保持するものとする。

**12.研究結果の帰属と発表**

・ICMJEの勧告に基づき、UMIN等に臨床試験登録を行う。

・結果の公表の際には、生物統計家、データマネージャーを共同研究者に加える。

・登録症例数の順による著者順位の規定やcorresponding authorの規定を事前に行う。

**13.付録**

pain scale　及び　記録用紙

　添付ファイル

説明文書・同意書

添付ファイル
