# Supplementary material for: A Randomized, Single-Blind, Placebo-Controlled Study on the Efficacy of the Arthrokinematic Approach-Hakata Method in Patients with Chronic Nonspecific Low Back Pain
Source: PLoS One. 2015 Dec 8;10(12):e0144325. doi: 10.1371/journal.pone.0144325 (PMC4672908; doi:10.1371/journal.pone.0144325)
Supplement: S1 Table — Data are shown as mean ± standard deviation. VAS, visual analogue scale; RDQ, Roland-Morris Disability Questionnaire; SF-36, 36-Item Short-Form Health Survey; AKA-H, Arthrokinematic approach Hakata method. Significant difference (two-way [time period and group] repeated measures analysis of variance): * P < 0.05, ** P < 0.01. The AKA-H vs. sham group using Bonferroni multicomparison tests, † P < 0.008, †† P < 0.0016. (DOCX) [file pone.0144325.s009.docx]

Table 2. The levels of the visual analogue scale, Roland-Morris Disability Questionnaire, and 36-Item Short-Form Health Survey at 1 month to 6 months after treatment [LOCF]

|  | 1 month | | 2 months | | 3 months | | 4 months | | 5 months | | 6 months | | P-value  [time and  group] |
| --- | --- | --- | --- | --- | --- | --- | --- | --- | --- | --- | --- | --- | --- |
|  | AKA-H group  (n = 93) | Sham group  (n = 93) | AKA-H group  (n = 93) | Sham group  (n = 93) | AKA-H group  (n = 93) | Sham group  (n = 93) | AKA-H group  (n = 93) | Sham group  (n = 93) | AKA-H group  (n = 93) | Sham group  (n = 93) | AKA-H group  (n = 93) | Sham group  (n = 93) |  |
| VAS (points) | 46.3 ± 20.6 | 47.6 ± 20.5 | 42.8 ± 20.2 | 47.8 ± 21.1 | 36.3 ± 17.9 ^††^ | 47.6 ± 21.3 | 35.5 ± 17.5 ^††^ | 46.6 ± 22.1 | 32.6 ± 17.6 ^††^ | 45.8 ± 21.9 | 30.8 ± 18.7 ^††^ | 45.8 ± 21.9 | <0.001** |
| RDQ (deviation score) | 41.9 ± 10.9 | 40.9 ± 9.4 | 43.5 ± 10.7 | 41.3 ± 9.7 | 44.9 ± 10.2 | 41.5 ± 9.7 | 47.1 ± 9.1 ^††^ | 42.2 ± 10.1 | 47.8 ± 9.6 ^††^ | 42.3 ± 9.8 | 49.3 ± 9.1 ^††^ | 42.3 ± 9.9 | <0.001** |
| SF-36 (points) |  |  |  |  |  |  |  |  |  |  |  |  |  |
| Physical functioning | 33.6 ± 14.2 | 31.9 ± 13.3 | 35.9 ± 13.0 | 31.0 ± 13.3 | 38.1 ± 12.9 ^††^ | 31.4 ± 13.3 | 40.2 ± 12.6 ^††^ | 32.2 ± 13.7 | 41.2 ± 13.5 ^††^ | 32.3 ± 13.9 | 42.0 ± 13.9 ^††^ | 32.5 ± 14.2 | <0.001** |
| Role physical | 32.0 ± 12.8 | 32.6 ± 13.2 | 34.8 ± 12.6 | 32.6 ± 13.4 | 36.2 ± 13.2 | 32.0 ± 14.5 | 38.8 ± 12.8 ^††^ | 32.0 ± 14.4 | 40.7 ± 13.0 ^††^ | 32.4 ± 13.5 | 40.6 ± 13.7 ^††^ | 32.3 ± 13.5 | <0.001** |
| Bodily pain | 35.0 ± 7.3 | 33.9 ± 6.8 | 37.3 ± 7.4 ^†^ | 33.9 ± 7.3 | 38.1 ± 8.4 ^††^ | 34.1 ± 7.1 | 40.1 ± 8.1 ^††^ | 34.4 ± 7.4 | 41.5 ± 9.1 ^††^ | 34.2 ± 7.4 | 42.4 ± 9.8 ^††^ | 33.4 ± 7.7 | <0.001** |
| Social functioning | 38.1 ± 12.7 | 35.3 ± 12.0 | 40.6 ± 12.8 ^†^ | 35.5 ± 11.9 | 43.1 ± 11.8 ^††^ | 33.9 ± 12.4 | 45.9 ± 11.2 ^††^ | 34.7 ± 12.3 | 46.3 ± 11.2 ^††^ | 35.7 ± 12.1 | 48.3 ± 10.6 ^††^ | 34.5 ± 12.7 | <0.001** |
| General health perception | 40.2 ± 9.8 | 39.5 ± 6.5 | 40.6 ± 9.8 | 39.7 ± 6.6 | 42.6 ± 8.7 ^†^ | 39.2 ± 7.3 | 43.3 ± 9.2 ^†^ | 39.5 ± 7.9 | 43.8 ± 9.0 ^††^ | 39.3 ± 7.4 | 44.6 ± 9.8 ^††^ | 39.2 ± 7.8 | <0.001** |
| Vitality | 42.0 ± 8.6 | 42.3 ± 9.0 | 43.9 ± 9.1 | 42.3 ± 9.2 | 45.9 ± 9.1 ^†^ | 41.4 ± 10.0 | 46.8 ± 9.1 ^††^ | 41.3 ± 10.3 | 47.5 ± 9.2 ^††^ | 41.0 ± 10.0 | 47.8 ± 9.6 ^††^ | 41.4 ± 9.9 | <0.001** |
| Role emotional | 37.6 ± 13.3 | 37.7 ± 11.2 | 40.2 ± 11.8 | 37.1 ± 11.1 | 41.7 ± 11.6 ^†^ | 36.6 ± 12.6 | 44.8 ± 10.9 ^††^ | 36.6 ± 12.1 | 45.3 ± 11.9 ^††^ | 36.8 ± 11.8 | 45.8 ± 11.8 ^††^ | 36.0 ± 11.9 | <0.001** |
| Mental health | 43.7 ± 9.1 | 42.5 ± 8.4 | 45.9 ± 9.8 ^†^ | 41.8 ± 8.3 | 47.7 ± 9.4 ^††^ | 42.2 ± 8.6 | 48.9 ± 9.0 ^††^ | 41.7 ± 9.0 | 48.8 ± 10.5 ^††^ | 41.9 ± 9.5 | 49.8 ± 9.6 ^††^ | 42.2 ± 8.7 | <0.001** |

Data are shown as the mean ± SD.

VAS, Visual analogue scale; RDQ, Roland-Morris Disability Questionnaire; SF-36, 36-Item Short-Form Health Survey; AKA-H, Arthrokinematic approach Hakata method.

Significant difference (two-way [time period and group] repeated measures analysis of variance): * P < 0.05, ** P < 0.01

The AKA-H vs. sham group using Bonferroni multicomparison tests, †P < 0.008, ††P < 0.0016.
